# Pseudoangiomatous Stromal Hyperplasia: A Case Report

**DOI:** 10.1155/2010/549643

**Published:** 2011-01-24

**Authors:** Yazan A. Masannat, Stephen Whitehead, Ian Hawley, Lesley Apthorp, Elizabeth F. Shah

**Affiliations:** ^1^Department of General Surgery, Princess Royal University Hospital, Farnborough Common, Orpington BR6 8ND, UK; ^2^Breast Unit, Conquest Hospital, The Ridge, St. Leonards-on-Sea, East Sussex TN37 7RD, UK

## Abstract

Pseudoangiomatous stromal hyperplasia (PASH) is a rare benign proliferating breast condition. It was first reported in 1986 when Vuitch, Rosen, and Erlandson described nine cases of benign well-circumscribed, breast masses that simulated vascular lesions consisting of mammary stromal proliferations (Vuitch et al. (1986)). Since then there have been few reported cases of PASH in the literature (Taira et al. (2005)). We describe a large PASH, mimicking inflammatory carcinoma in a young lady that was excised with excellent cosmetic results.

## 1. Case Report

**Figure 1:**
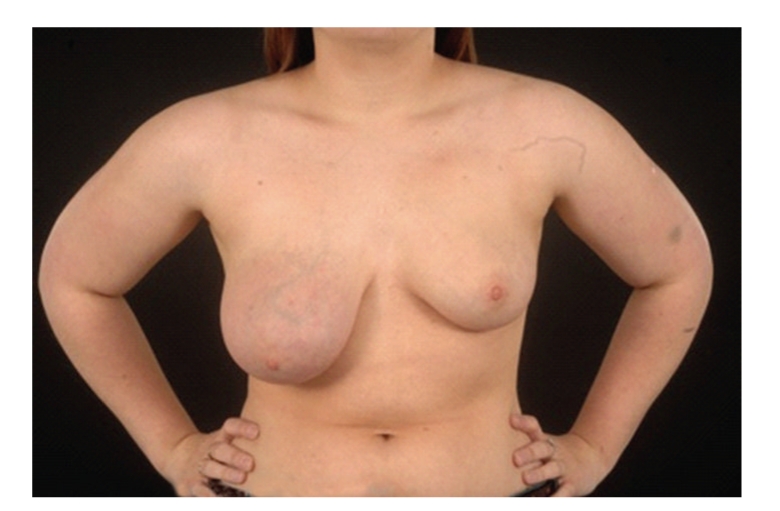
Figure demonstrating the asymmetry and the large mass in the right breast on presentation.

**Figure 2:**
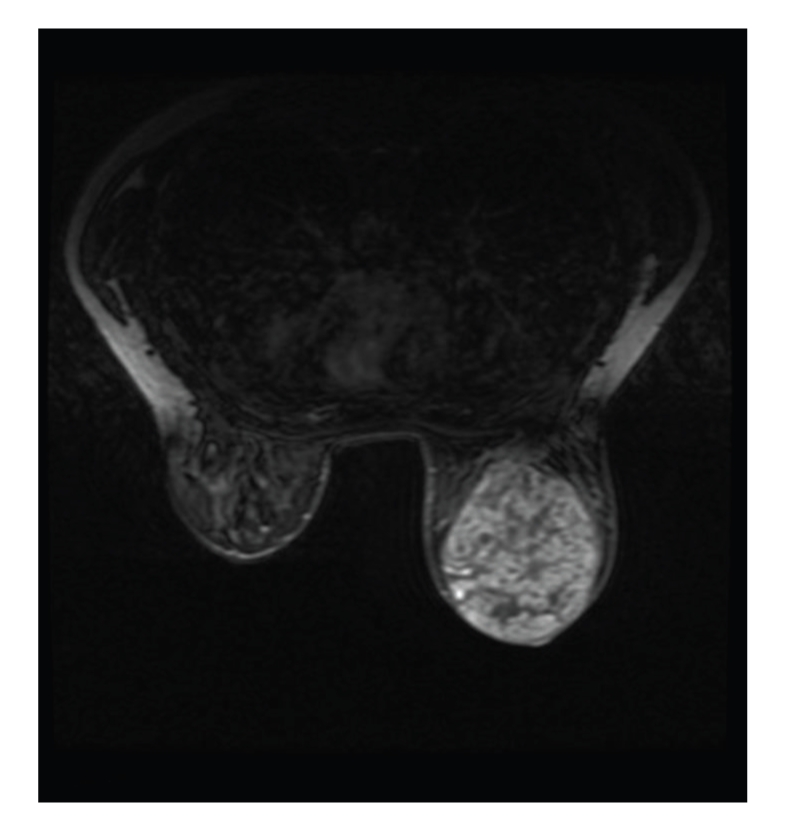
MRI showing the 10 cm well-circumscribed rounded mass lesion which demonstrated a benign enhancement pattern after intravenous Gadolinium.

A 22-year-old lady presented to the breast clinic with a one-year history of a lump in the right breast. The lump progressively increased in size until there was a marked difference between both breasts. On clinical assessment, the right breast was diffusely erythematous with prominent superficial veins and skin changes. The palpable mass was around 10 × 8 cm in diameter and felt clinically suspicious of malignancy mimicking inflammatory carcinoma (Figure 1). Ultrasound scan showed a 10 × 5 cm mass consistent with fibroadenoma or Phyllodes tumour, while MRI showed a 10 cm well-circumscribed rounded mass lesion which demonstrated a benign enhancement pattern after intravenous Gadolinium. Radiologically the appearances remained most suggestive of a fibroadenoma or Phyllodes tumour, but also in keeping with PASH (Figure 2).

**Figure 3:**
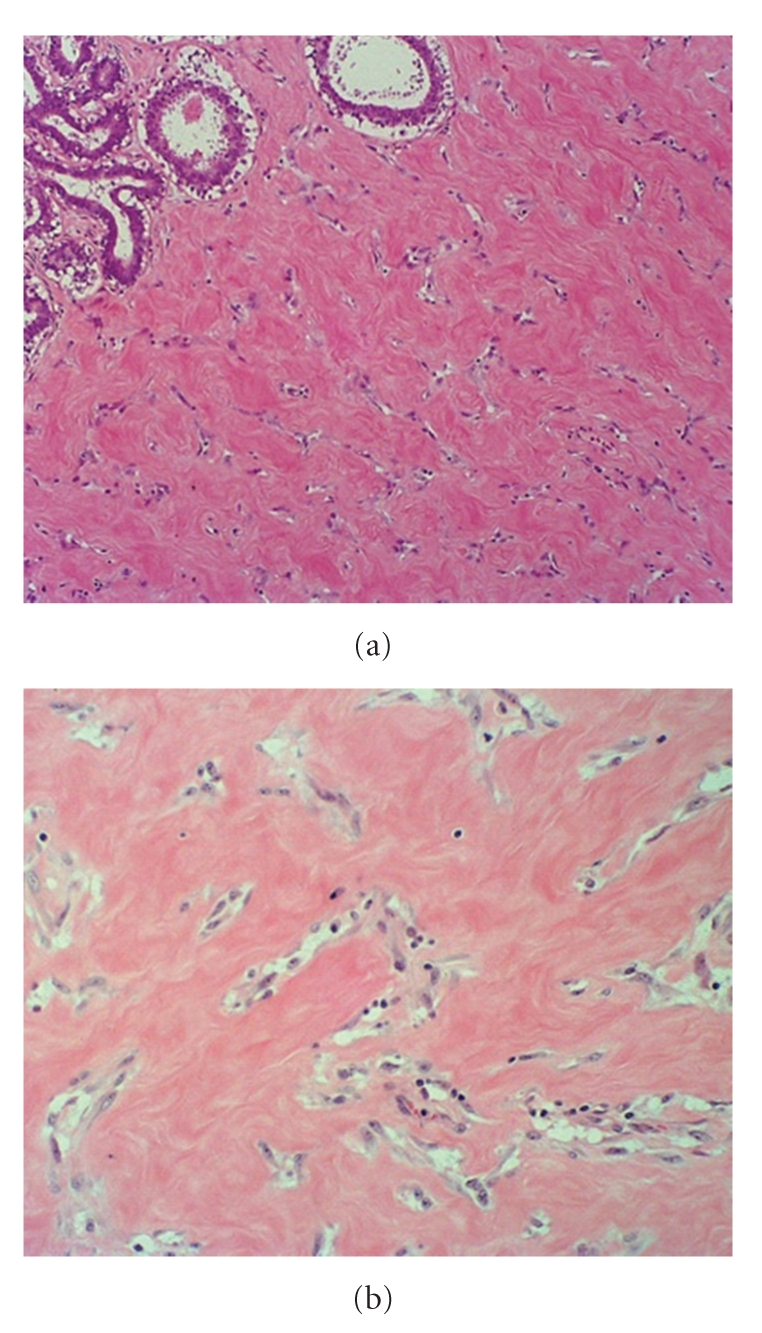
(a) Anastomosing groups of spindle cells in the stroma. Normal breast epithelium top left. (b) Stromal spindle cells which appear to be lining slit-like spaces devoid of red blood cells.

 Fine needle aspiration cytology results were equivocal showing cohesive cells with focal mild nuclear atypia and numerous bare nuclei. Core biopsy was done that showed the presence of spindle cells in the fibrous tissue in a pseudoangiomatous pattern which fitted the diagnosis of PASH. This was the same finding on the final histopathology after excision (Figure 3). Immunohistochemistry was positive for CD34 and Vimentin while Factor VIII, cytokeratin, progesterone, and oestrogen receptors were negative. 

**Figure 4:**
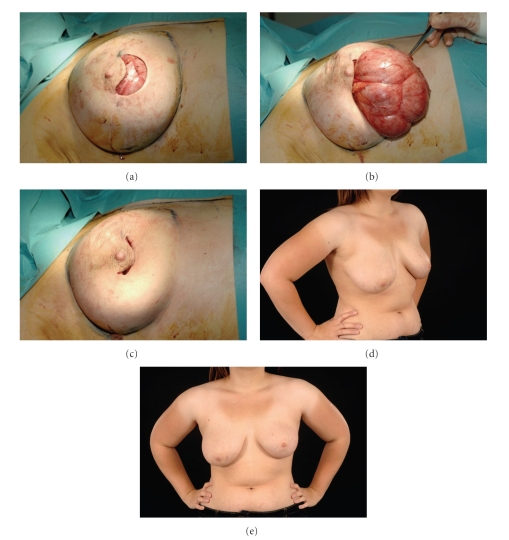
((a), (b), and (c)) Intraoperative pictures. (d) Postoperative pictures show the good cosmetic results with slight asymmetry between both sides, reported by the patient as being her normal as before the PASH appeared.

Surgical excision was done using an inferior circumareolar incision. The mass was excised through a small incision, and the breast with simple glandular remodelling the patient had excellent cosmetic results postoperatively (Figure 4). The patient has been followed up for 18 months now without evidence of recurrence.

## 2. Discussion

PASH is a benign proliferation of the mammary stromal tissue. Histologically it shows complex interanastomosing slit-like spaces which appear to be lined by spindle cells in the breast parenchyma [1]. This is not an uncommon finding in both benign and malignant breast specimens with up to 23% of the specimens showing small foci of PASH [2]. It is, however, rare for a discrete mass to have PASH as the main pathological feature on histopathology with only around a hundred cases reported in the literature. 

These tumours are classified into either simple or fascicular/proliferative subtypes. In the simple type open, slit-like anastomosing channels without erythrocytes appear to be lined by flat cells in a discontinuous layer while in the fascicular/proliferative type there are areas of cellular proliferation composed of bland spindle cells. One paper describes gynaecomastia-like changes in nearly two thirds of the cases [3]. It is important to differentiate the lesion from low-grade angiosarcoma. PASH does not exhibit any atypia or mitotic activity, and there are no blood cells seen in the slit-like structures [4]. Cytology is nonspecific and it is impossible to diagnose on fine needle aspiration cytology. Core biopsy or Mammotome biopsy is needed for diagnosis preoperatively, although in some cases the final diagnosis is made only after excision [4]. On immunohistochemistry, PASH is positive for CD34 and vimentin and negative for factor VIII-related antigen and cytokeratin [3–5].

On radiology there are no specific or diagnostic features. Most of these tumours mimic fibroadenomas or hamartomas though other sinister pathologies such as angiosarcomas come into the differential diagnosis and larger tumours can be mistaken for Phyllodes tumour [3, 6, 7]. On mammography the commonest finding is a well-defined mass while ultrasound scan shows usually a hypoechoic discrete mass with benign features [8, 9] though some have described cases where the mass was either spiculated, irregular or had ill-defined margins on radiology [3, 6].

The aetiology of this is not well understood though some authors have suggested a hormonal cause of this condition [1, 4, 10]. Many of the patients diagnosed are premenopausal, and there is association with the use of hormones for contraception or as hormonal replacement therapy [3, 10]. Many have reported positive progesterone receptor status in those lesions which further supports this theory but our case was negative for both oestrogen and progesteron receptors [4, 5]. Pruthi et al. reported treating one patient with Tamoxifen with good results [11].

If diagnosis is confirmed on biopsy and the lesion is small and asymptomatic, then surgical excision can be avoided but for the larger lesions, and especially if there are suspicious features clinically or radiologically, then surgical excision is indicated [3, 4]. While most will perform an excision biopsy to confirm the diagnosis ad get clear margins, some have performed mastectomies especially for the larger lesions. In our report, though the lesion was large, it was still all excised through as small circumareolar incision. Symmetrisation procedure was considered for this particular case because of the size difference before excising the lesion, but following the excision the patient was happy with the cosmetic result as the size and shape of the operated breast was reported to be back to normal as before the PASH appeared. 

PASH is a rare benign condition that is diagnosed on triple assessment. If it is proven on biopsy, then regular followup is suitable. Surgery is reserved for people with large lesions or if the histology did not confirm the diagnosis preoperatively and if there is any suspicious features on triple assessment or patients choice. Followup following excision is recommended as local recurrence has been reported.
